# 59. Risk Factors for Recurrent Gram-Negative Bacterial Bloodstream Infections

**DOI:** 10.1093/ofid/ofab466.059

**Published:** 2021-12-04

**Authors:** Andrew J Bock, Batu K Sharma-Kuinkel, Felicia Ruffin, Michael Mohnasky, Emily Eichenberger, Stacey Maskarinec, Vance G Fowler, Joshua Thaden

**Affiliations:** 1 Duke University School of Medicine, Dawsonville, GA; 2 Duke University Medical Center, Durham, NC; 3 Duke University, Durham, North Carolina

## Abstract

**Background:**

Gram-negative bacteria bloodstream infections (GNB-BSI) are a significant cause of morbidity and mortality. Recurrent GNB-BSI is an incompletely understood phenomenon. In this study we identify risk factors for recurrent GNB-BSI.

**Methods:**

Patients with GNB-BSI have been prospectively enrolled into the Bloodstream Infection Biorepository (BSIB) since 2002. From the BSIB, patients with >1 episode of GNB-BSI with the same bacterial species were identified. Chi-Square, Fisher Exact, and a multivariate linear regression models were used to identify clinical risk factors for recurrent GNB-BSI. Paired isolate samples from the initial and the recurrent episode of GNB-BSI in same patient underwent Pulsed Field Gel Electrophoresis (PFGE) to differentiate ***Relapse ***(paired isolates identical) from ***Reinfection ***(paired isolates different).

**Results:**

Among the 1,423 unique patients with GNB-BSI enrolled from 2002- 2015, 60 (4.2%) experienced recurrent GNB-BSI with the same bacterial species. Median time to recurrent GNB-BSI was 133 d (IQR: 40-284.75 days). Causes of recurrent-GNB-BSI included

*Escherichia coli (38%), Klebsiella species (30%), Pseudomonas aeruginosa (12%), *and *Serratia marcescens (5%) and did not differ from causes of non-recurrent GNB-BSI (Figure 1). *Risk factors for recurrent GNB-BSI included Black race (OR: 2.45 [CI: 1.43-4.20]), implanted cardiac device (OR: 2.39 [CI: 1.00-5.07]), and admission to surgical service (OR: 2.16 [CI 1.24-3.75]). Forty-eight isolate-pairs from 43 patients with recurrent GNB-BSI underwent PFGE, relapse occurred in 31 (65%) and reinfection occurred in 17 (35%). Risk factors for GNB-BSI relapse included cardiac device (OR: 3.7 [CI: 1.7-8.3]), and admission to surgical service (OR: 3.7 [CI:1.3-9.4]).

Figure 1: Species Breakdown

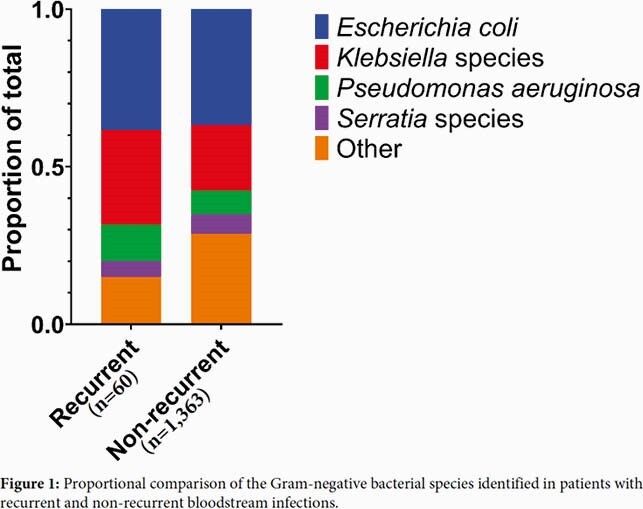

Proportional comparison of the Gram-negative bacterial species identified in patients with recurrent and non-recurrent bloodstream infections.

**Conclusion:**

Recurrent GNB-BSI is an uncommon complication of GNB-BSI. Recurrent GNB-BSI is most often driven by relapse, as opposed to reinfection, and is associated with associated with black race, implanted cardiac devices and admission to surgical service.

**Disclosures:**

**Vance G. Fowler, Jr., MD, MHS**, **Achaogen** (Consultant)**Advanced Liquid Logics** (Grant/Research Support)**Affinergy** (Consultant, Grant/Research Support)**Affinium** (Consultant)**Akagera** (Consultant)**Allergan** (Grant/Research Support)**Amphliphi Biosciences** (Consultant)**Aridis** (Consultant)**Armata** (Consultant)**Basilea** (Consultant, Grant/Research Support)**Bayer** (Consultant)**C3J** (Consultant)**Cerexa** (Consultant, Other Financial or Material Support, Educational fees)**Contrafect** (Consultant, Grant/Research Support)**Debiopharm** (Consultant, Other Financial or Material Support, Educational fees)**Destiny** (Consultant)**Durata** (Consultant, Other Financial or Material Support, educational fees)**Genentech** (Consultant, Grant/Research Support)**Green Cross** (Other Financial or Material Support, Educational fees)**Integrated Biotherapeutics** (Consultant)**Janssen** (Consultant, Grant/Research Support)**Karius** (Grant/Research Support)**Locus** (Grant/Research Support)**Medical Biosurfaces** (Grant/Research Support)**Medicines Co.** (Consultant)**MedImmune** (Consultant, Grant/Research Support)**Merck** (Grant/Research Support)**NIH** (Grant/Research Support)**Novadigm** (Consultant)**Novartis** (Consultant, Grant/Research Support)**Pfizer** (Grant/Research Support)**Regeneron** (Consultant, Grant/Research Support)**sepsis diagnostics** (Other Financial or Material Support, Pending patent for host gene expression signature diagnostic for sepsis.)**Tetraphase** (Consultant)**Theravance** (Consultant, Grant/Research Support, Other Financial or Material Support, Educational fees)**Trius** (Consultant)**UpToDate** (Other Financial or Material Support, Royalties)**Valanbio** (Consultant, Other Financial or Material Support, Stock options)**xBiotech** (Consultant)

